# Supplementary material for: Patterns of Intron Gain and Loss in Fungi
Source: PLoS Biol. 2004 Nov 30;2(12):e422. doi: 10.1371/journal.pbio.0020422 (PMC532390; doi:10.1371/journal.pbio.0020422)
Supplement: Table S1 — Also available at http://genes.mit.edu/NielsenEtAl/. (4.3 MB ZIP). [file pbio.0020422.st001.zip › NielsenEtAl/html/1337.html]

AN6048.1.NCU07941.1.MG04156.1.FG09739.1


```
 CLUSTAL W (1.82) Multiple Sequence Alignments - Introns Inserted


Sequence 1: NCU07941.1	482 aa
Sequence 2: FG09739.1	414 aa
Sequence 3: MG04156.1	457 aa
Sequence 4: AN6048.1	445 aa
Alignment Length: 484 aa
Number Identitical Residues: 244 aa
Alignment Score (without introns) 11623


MG04156.1 	--------------------MSAKATRGSTSSSTPQNSSSSSQSQSQSTAVSRIRNIANH
NCU07941.1	MAPASTLLRAAAPAPHAHTSFPSHHTPTSSPNRTTITNTNPSANSTSSTPLHRIQTIAKH
FG09739.1 	------------------------------------------------------------
AN6048.1  	-----------------------------------MSTPSSVSSSSYSAAKSRLASLASH
          	                                    .....  ..: ::.     . :. 

MG04156.1 	MMASPSTI--FTADVVPQAPEDPLFGLMAAYRADESPDKVDL0GIGAYRDDNAKPWVLPV
NCU07941.1	MALPQITS--FPAEVVPQAPEDPLFGLARAFKADPSPQKVDL0GIGAYRDENAKPWVLPV
FG09739.1 	--MVSTTN--FPAEVVPQAPEDPLFGLAREYKADDSPNKIDL0GIGAYRDENAKPWVLPV
AN6048.1  	IMGSSSASPVFSTAVVPSAPEDPLFGLAQAYRQDPSDKKVDL~VIGAYRDDNAKPWVLPV
          	      : . *.: ***.*********   :: * * .*:**  ******:*********

MG04156.1 	VKK0ADEIIRNDPSANHEYLPITGLASFTSKAGELMLGADTPAK--GRVTSVQTISGTGA
NCU07941.1	VKK0ADEIIRNDPEANHEYLPIAGLASLTSKAAELVVGASAPAITEGRVASIQTISGTGA
FG09739.1 	VKK0ADEILRNDPELNHEYAPIAGIASFTSKAAELVFGPDSSAIQDKRSTTLQTISGTGA
AN6048.1  	VKK0ADELIRNDPNLNHEYLPIKGLAEYTTAAQKLIIGADSPAIAENRVCTFQTISGTGA
          	*** ***::****. **** ** *:*. *: * :*:.*..:.*  . *  :.********

MG04156.1 	LHLGALFLQKFYR---KVYSNSVVHLSNPTWANHNQIFSNVQVPTTTYPYFDKGTKGLDF
NCU07941.1	CHLGGLFLSRFYNPYGDASKKPTVYLSNPTWANHNQIFSNVGLPIAQYPYFDQKTKGLDI
FG09739.1 	VHLGALFLAKFYK------GNQTVYLSNPTWANHHQIFKNVGMSIDTYPYFHKETKGLDF
AN6048.1  	VHLGALFLARFHP----ATPKPTLYLSSPTWANHHQIFTNVGFTLANYPYFSPQTKGLDF
          	 ***.*** :*:        : .::**.******:***.** ..   ****   *****:

MG04156.1 	EGMKATLNNAAEHSIILLHACAHNPTGVDPTQGQWREIAEIMKAKKHFPFFDCAYQGFAS
NCU07941.1	DGMKKALSDAPERSIILLHACAHNPTGVDPTLAQWREIAEIMAAKGHFPFFDTAYQGFAS
FG09739.1 	EGFKKTLQSAPEGSVFVLHACAHNPTGVDPTQDQWTEIASIMKEKNHFPFFDTAYQGFAS
AN6048.1  	DGMINALRSAPAGSIILLHACAHNPTGVDLTQEQWKEVAVVMRDRSHFPFFDCAYQGFAS
          	:*:  :* .*.  *:::************ *  ** *:* :*  : ****** *******

MG04156.1 	GDLDRDAWAVRYFVEQGFELVIAQSFAKNFGLYGERAGCFHYVSAPAAEAAETTKRVASQ
NCU07941.1	GDLDRDASAIRLFVELGFELVVAQSFAKNFGLYGQRAGCFHFISAPSPDAASITTRVASQ
FG09739.1 	GDLVKDAWAIRYFVDQGFELVVAQSFAKNFGLYGERAGCFHAVTSPAPEASNTITRIGSQ
AN6048.1  	GDLARDAWAIRYFVEQGFELCIAQSFAKNFGLYGERTGAFHFVSAPGPEAAQSSAHVASQ
          	*** :** *:* **: **** :************:*:*.** :::*..:*:.   ::.**

MG04156.1 	LAILQRSEISNPPIYGARVASIVLNDPALMSEWRENLRTMSGRIITMRNELRAKLEALGT
NCU07941.1	LTLLQRSEISNPPIYGAKVASIVLNDPALFAEWKENLRTMSGRIIDMRKALRAKLEELGT
FG09739.1 	LAILQRSEISNPPLYGARIVSTVLNDRDLFAEWEENLRTMSGRIISMRDVLRAKLEELET
AN6048.1  	LAILQRSEISNPPAYGARIASKVLNDPELFAQWEDDLRTMSGRIVEMRKGLRQRLEEKKT
          	*::********** ***::.* ****  *:::*.::********: **. ** :**   *

MG04156.1 	PGTWNHITDQIGMFSFTGLTEAQVLKIRSDYHVYM~TKNGRISMAGLNSRNIDYFAKAVD
NCU07941.1	PGPWNHITDQIGMFSFTGLNEKQVAKLREEFHIYM~TKNGRISMAGLNKKNVDYVARAVD
FG09739.1 	PGTWNHITDQIGMFSFTGLSEKQVLQLREEFHVYM~TKNGRISMAGLNENNVEYFAKAVD
AN6048.1  	PGSWNHITDQIGMFSFTGLSEAQVKVLREKWHVYM0TKNGRISMAGLNSHNLDYFAEAVD
          	**.****************.* **  :*..:*:** ************..*::*.*.***

MG04156.1 	KVVRETQ
NCU07941.1	KVVREVQ
FG09739.1 	KVVRDAA
AN6048.1  	SVVRETS
          	.***:.
```
